# Update on Abdominal Aortic Aneurysm Research: From Clinical to Genetic Studies

**DOI:** 10.1155/2014/564734

**Published:** 2014-04-17

**Authors:** Helena Kuivaniemi, Evan J. Ryer, James R. Elmore, Irene Hinterseher, Diane T. Smelser, Gerard Tromp

**Affiliations:** ^1^The Sigfried and Janet Weis Center for Research, Geisinger Health System, Danville, PA, USA; ^2^Department of Surgery, Temple University School of Medicine, Philadelphia, PA, USA; ^3^Department of Vascular and Endovascular Surgery, Geisinger Health System, Danville, PA, USA; ^4^Department of General, Visceral, Vascular and Thoracic Surgery, Charité Universitätsmedizin Berlin, Charité Campus Mitte, Berlin, Germany

## Abstract

An abdominal aortic aneurysm (AAA) is a dilatation of the abdominal aorta with a diameter of at least 3.0 cm. AAAs are often asymptomatic and are discovered as incidental findings in imaging studies or when the AAA ruptures leading to a medical emergency. AAAs are more common in males than females, in individuals of European ancestry, and in those over 65 years of age. Smoking is the most important environmental risk factor. In addition, a positive family history of AAA increases the person's risk for AAA. Interestingly, diabetes has been shown to be a protective factor for AAA in many large studies. Hallmarks of AAA pathogenesis include inflammation, vascular smooth muscle cell apoptosis, extracellular matrix degradation, and oxidative stress. Autoimmunity may also play a role in AAA development and progression. In this Outlook paper, we summarize our recent studies on AAA including clinical studies related to surgical repair of AAA and genetic risk factor and large-scale gene expression studies. We conclude with a discussion on our research projects using large data sets available through electronic medical records and biobanks.

## 1. Introduction

An abdominal aortic aneurysm (AAA) is a permanent localized dilatation of the abdominal aorta with a transverse diameter ≥3.0 cm [1]. AAAs are often asymptomatic and are discovered as incidental findings in imaging studies (e.g., abdominal ultrasonography or computerized tomography (CT) examination) or when the AAA ruptures leading to a medical emergency. AAAs are more common in males than females (6 : 1 ratio), in individuals of European ancestry and in those >65 years of age [2–8]. Smoking is the most important environmental risk factor for AAA development [2–8]. In addition, a positive family history of AAA increases a person's risk for AAA [2–9]. Interestingly, diabetes has been shown to be a protective factor for AAA in many large studies [2–8].

Hallmarks of AAA pathogenesis include inflammation, vascular smooth muscle cell (VSMC) apoptosis, extracellular matrix (ECM) degradation, and oxidative stress [2, 5, 6, 10]. In AAA patients, T-cells secrete proinflammatory cytokines [11] and natural killer cells display increased cytotoxicity [12]. Autoimmunity may also play a role in AAA development and progression [3, 13].

In this Outlook paper, we summarize our recent studies on AAA starting with clinical observations and then moving into studies on the genetic risk factors for AAA followed by studies on the pathogenesis of AAA, large-scale gene expression studies and studies on animal models. Finally, we discuss future research using large data sets available through electronic medical records (EMR) and biobanks.

## 2. Clinical Studies on AAA

### 2.1. Rupture of AAA Is Preventable

The main risk of an untreated AAA is progressive expansion, rupture, hemorrhage, and death. AAA prevalence increases with age, and even though the incidence has declined in the past ten years [14], rupture of AAA is still a significant cause of death with 10–15% of AAAs presenting as ruptured AAAs (rAAA) in the emergency room. AAA rupture risk increases with increasing aortic diameter and this catastrophic event is associated with a mortality of 50 to 80% [15–17]. Due to the excessive mortality associated with emergent repair, the mainstay of AAA management is early diagnosis and elective repair prior to rupture. In an effort to reduce the number of AAA-related deaths, the Screening Abdominal Aortic Aneurysms Very Efficiently (SAAAVE) Act was approved by the United States Congress in January 2007. The SAAAVE Act permits a single screening aortic ultrasound examination as part of the “Welcome to Medicare” package for patients with defined risk factors for AAA. Specifically, males between the age of 65 and 75 years who have smoked greater than 100 cigarettes in their lifetime or patients of any age or sex with a strong family history are eligible for this screening examination [18]. Despite the introduction of the SAAAVE Act in 2007, screening for AAA remains underutilized [19]. Additionally, more recent data suggest that the implementation of the SAAAVE Act has not had any discernible effect on AAA rupture rate or AAA-related mortality, presumably due to its severe underutilization [20].

We have an active interest in AAA screening and studied whether routine medical evaluation in the ambulatory care setting combined with the utilization of the SAAAVE Act, in its current form, is an effective method for screening and diagnosing patients at risk for AAA [21]. To test our hypothesis, we retrospectively reviewed 149 consecutive patients presenting to our tertiary care center, Geisinger Health System (GHS) [22], with a rAAA. Of these 149 patients, 52 patients had complete EMR documentation of an ambulatory care visit within the 6 months prior to the date of rupture. Twenty-one of the ruptures (40%) had a radiographic study (duplex ultrasound or CT scan) that demonstrated an AAA. Only 12 of these 21 (57%) were referred to and evaluated by a vascular surgeon. Furthermore, under the current SAAAVE Act criteria, only 9/52 (17%) of the study patients would have been eligible for the “Welcome to Medicare” screening aortic ultrasound (Table 1). Of the male patients ineligible for screening under the SAAAVE Act, 7/36 (14%) were younger than 65 years and 19/36 (37%) were older than 75 years. Only 10/36 (19%) male patients were between the age of 65 and 75 years, seven of which were eligible for the screening ultrasound secondary to tobacco use. Eleven of the 19 male patients older than 75 years had a history of smoking and would have qualified under more inclusive age criteria. There were also 16/52 (31%) females between the age of 61 and 96 years who presented with a rAAA. Thirteen (81%) of the females had a smoking history. Unfortunately, AAA screening in female patients is not covered by the SAAAVE Act. Overall, greater than 80% of patients who presented to our institution with a rAAA would not be included in the SAAAVE Act screening program. From our investigation, we concluded that routine ambulatory medical evaluations in conjunction with current AAA screening methods are inadequate to detect AAAs in an at-risk population prior to rupture.

### 2.2. Quality of Life for AAA Patients

We have also examined the long-term quality of life (QOL) in AAA patients under surveillance and after operative treatment [23]. We performed a retrospective analysis of 78 patients with small aneurysms (diameter 3.0–4.9 cm) undergoing surveillance at University Hospital of Dresden in Germany and compared them with long-term survivors following operative treatment during the time period of 1995 to 2006. The surgical group included 26 patients undergoing open repair for the treatment of a rAAA, 47 patients following elective endovascular aneurysm repair (EVAR), and 98 patients following elective open repair. Patients were assessed using the WHO Quality of Life-BREF (WHOQOL-BREF) and Short Form Health Survey (SF-36). The WHOQOL-BREF is an internationally standardized QOL questionnaire that exists in different languages and is proven to determine reduced QOL in chronic diseases and the SF-36 is a well-known international QOL test, often used after surgical procedures. SF-36 is also available in multiple languages. Surprisingly, we found that patients undergoing surveillance for a small AAA had significantly diminished physical health (*P* = 0.04) scores on the WHOQOL-BREF and significantly decreased role-emotional (*P* = 0.003) and role-physical (*P* = 0.02) on the SF-36 compared to the age- and sex-matched German control subjects. In contrast, patients undergoing elective AAA repair had similar QOL scores as matched German subjects. It is likely that the lower QOL in AAA patients undergoing surveillance can be improved through more intensive education about their health condition and the low risk of rupture in the early stages of the disease. Further studies will be needed to see if our efforts will improve QOL in small AAA patients undergoing surveillance.

### 2.3. Survival Rates of AAA Patients after Operative Treatment

**Figure 1:**
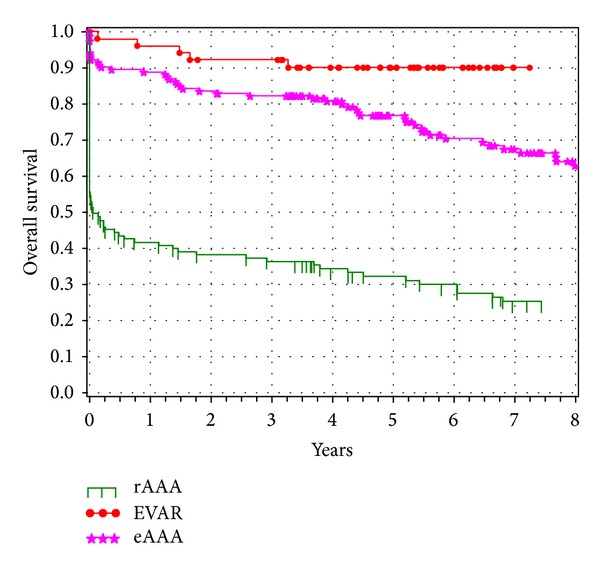
Survival analysis for patients after a ruptured abdominal aortic aneurysm (rAAA), elective open repair (eAAA), or elective endovascular repair (EVAR). Kaplan-Meier curves were generated with the indicated numbers of patients at risk. Reproduced with permission from [26].

Once AAAs are identified, they can be managed using different therapeutic modalities: traditional open surgical repair, minimally invasive EVAR, or continued surveillance. Treatment in most cases is selected based on aneurysm size, aneurysm shape, and the patient's corresponding medical comorbidities. Traditional open surgical repair involves a large incision in the abdomen and replacement of the aneurysm with a synthetic fabric graft. An alternative treatment, first described in 1986, is EVAR [24]. This minimally invasive technique involves insertion of a fabric covered stent to reline the aneurysmal aorta via the femoral and iliac arteries. The function of the stent graft is to exclude the weakened aortic aneurysm wall from the systemic blood pressure and hence rupture. The introduction of EVAR has revolutionized the therapeutic approach to patients with AAA. Furthermore, the last decade has witnessed a rapid progression in the techniques and devices engineered to perform endovascular AAA stent grafting in an attempt to decrease graft-related complications and reinterventions. In our experience, suitable anatomy, device familiarity, and cost are the most important factors regarding selection for EVAR. In general, many of the current devices used according to instructions for use are equivalent regarding postoperative complications, migration, and aneurysm-related mortality [25]. In addition to device design, we have an active interest in studying the outcomes following AAA repair [26]. Recently, we compared the relative survival rates of patients after various operative treatments for AAA to those of the general population. To perform this study, we retrospectively reviewed the records of all patients who underwent open repair for rAAA, elective open repair, and elective EVAR of AAAs from 1995 to 2005 at the Vascular Center, Technical University of Dresden, Germany. The University Hospital of Dresden is among the higher-volume centers for AAA in Germany [27]. We studied 319 patients after operative treatment of AAA, including 113 (35%) with open rAAA repair, 152 (48%) with elective open AAA repair, and 54 (17%) with EVAR. Kaplan-Meier survival curves of the three operatively treated patient groups are shown in Figure 1. The rAAA patients showed a sharp decrease in survival during the first 6 months after operative management (including hospital mortality); 50% were alive after 6 months, 46% were alive after one year, and 36% were alive after three years. The elective open AAA repair group showed an 89% survival rate after one year and 82% after three years. The EVAR group had a survival rate of 96% after one year and 92% after three years. The time-specific relative survival rate of the rAAA group reached 1.0 at 16 months following emergency surgery and after ten months for open elective AAA repairs. The relative long-term survival rate in all three surgical groups was the same as that for the general German population. Our analysis demonstrated that the relative long-term survival after AAA repair, regardless of the modality, is equal to that of a comparable population-based group after the first postoperative month. We are hopeful that our efforts will assist clinicians in decision-making and may help a hesitant patient when deciding on management of their AAA.

### 2.4. Comparing the Cost of AAA Repair

**Figure 2:**
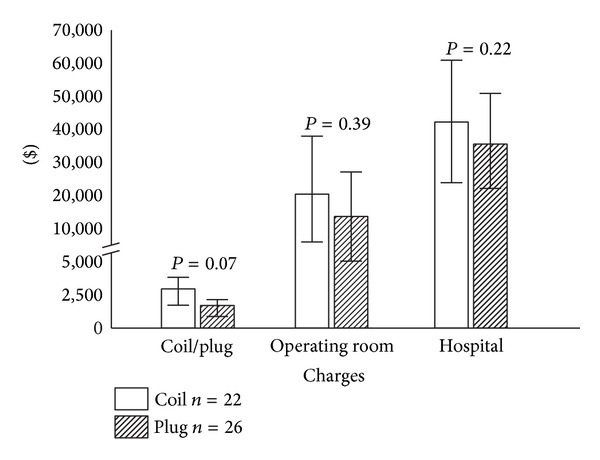
Economic analysis of internal iliac artery embolizations. Coil embolization (COIL) and Amplatzer Vascular Plug embolization (PLUG) charges, operating room charges, and total hospital charges for internal iliac artery embolizations performed prior to endovascular aneurysm repair for the time period from 2004 to 2010 (standardized to 2011 dollars). Reproduced with permission from [31].

In addition to studying clinical outcomes, we are also concerned with the cost of AAA repair. This is especially important as clinicians continually balance the use of emerging technology with efforts to decrease health care expenditures. Recent studies have shown that the standard minimally invasive EVAR procedure comes with decreased short term morbidity but also with increased costs [28–30]. Furthermore, EVAR commonly involves extending the aortic repair past the common iliac arteries into the external iliac arteries, which adds both complexity and expense. Without thrombosis of the excluded internal iliac artery (IIA), retrograde flow can lead to continued sac pressurization (i.e., endoleak) and aneurysm enlargement. Conventionally, embolization has been achieved safely by inserting embolization coils into the proximal IIA to induce thrombosis. The Amplatzer Vascular Plug (AGA Medical Corp, Plymouth, Minnesota), a nitinol-based self-expanding cylindrical occlusion device, has been shown to be a suitable alternative to coil embolization. Since both modalities have been shown to be successful techniques, and limited data directly comparing the two treatment modalities are available, we sought to compare the two techniques [31]. To do so, we performed a retrospective review of 53 consecutive patients (51 males and 2 females) treated with percutaneous IIA embolizations prior to EVAR from 2004 to 2010. Twenty-nine were treated with platinum embolization coils (Cook Medical Inc., Bloomington, Indiana) and 28 were treated with Amplatzer Vascular Plug devices. Patients undergoing Plug embolization demonstrated significantly shorter procedure times (*P* = 0.008); however, periprocedural success or morbidity did not differ between the groups. Furthermore, there was no significant difference in charges for the embolization material, operating room, or overall hospital charges (Figure 2). These findings supported our initial hypothesis that these two treatment modalities, despite differing expert opinions, are equal in terms of efficacy and cost.

## 3. The Discovery of New Genetic Risk Factors for AAA

AAA is a complex, multifactorial disease with a strong genetic component [4, 6–9, 32, 33]. About 20% of AAA patients have at least one relative with this condition [4, 6, 7, 9, 34]. Since the first candidate gene studies were published 20 years ago, nearly 100 genetic association studies using single nucleotide polymorphisms (SNPs) in biologically relevant genes have been reported on AAA [9]. More recently, unbiased genome-wide approaches such as family-based DNA linkage studies and genome-wide association studies (GWAS) have been carried out to identify susceptibility loci for AAA. By providing an updated synopsis of our AAA-related research efforts, we will examine the different approaches used to search out novel genetic risk factors.

### 3.1. DNA Linkage Studies

DNA linkage analysis is an unbiased genome-wide method used to identify susceptibility loci by following inheritance patterns of the disease of interest in a family through the use of genetic markers [35]. Typically, linkage between a specific genetic marker, or several markers, and the disease of interest can be determined using complex statistical analyses [36]. The most common approach of DNA linkage studies is to use large collections of multigenerational families. Due to the late-age-at onset of AAA and the fact that many patients die of ruptured AAA, it is difficult to collect blood samples for DNA isolations from large multigenerational families. DNA linkage studies on AAA have, therefore, used affected relative-pair approach. Using this approach two genetic loci, containing several plausible candidate genes located on chromosomes 4q31 and 19q13, were discovered for AAA [37–39].

### 3.2. AAA Genome-Wide Association Studies (GWAS)

GWAS uses a case-control approach and examines many common genetic variants in a large number of individuals to identify a variant that is associated with a specific trait. GWAS typically focuses on associations between SNPs, variations in a single nucleotide within the DNA sequence, and their influence on health and disease. If sufficiently large case-control groups are used, GWAS is an efficient method to identify reproducible risk alleles predisposing to the disease of interest [9, 40]. GWAS has proven a powerful instrument to identify genetic risk factors associated with the presence of cardiovascular disease and has been carried out by international consortia to identify susceptibility loci for AAA [28, 41–44].

The five chromosomal regions in the human genome with the strongest supporting evidence of contribution to the genetic risk for AAA are listed in Table 2 and include (1)* CDKN2BAS* gene (located on chromosome 9p21), also known as* ANRIL*, which encodes an antisense RNA that regulates expression of the cyclin-dependent kinase inhibitors* CDKN2A* and* CDKN2B* [44]; (2) DAB2 interacting protein (*DAB2IP*; located on chromosome 9q33), which encodes an inhibitor of cell growth and survival [43]; (3) low density lipoprotein receptor-related protein 1 (*LRP1*; located on chromosome 12q13.3), a plasma membrane receptor involved in vascular smooth muscle and macrophage endocytosis [45], (4) low density lipoprotein receptor (*LRPR*; located on chromosome 19p13.2) [41], and (5) contactin-3 (*CNTN3*; located on chromosome 3p12.3), which demonstrated the strongest association in smokers and yet its function remains unclear [42].

While the associations between these genetic loci and AAA are statistically strong, the functional variants contributing to the disease are not yet known. Furthermore, discovering the underlying biological mechanisms will require intense multidisciplinary efforts [46].

### 3.3. Pathway-Based Genetic Association Studies for AAA

As highlighted previously, GWAS is a powerful tool to detect genetic variants associated with a disease state of interest. With GWAS, it is common to focus on the statistically most significant results to avoid false positive results and ignore “less significant” variants with potentially greater biological plausibility. A pathway-based analysis approach to GWAS ranks differentially expressed genes by the significance of their expression along with the belief of plausibility and any prior disease association. In a recent investigation of ours [47], we applied such a biological pathway-based screen [48] to a whole-genome AAA case-control data set to determine whether variations in genes previously shown to be associated with either coronary artery disease (CAD) or blood lipids are also associated with AAA. The first phase of the study involved analyzing 44 lipid- and CAD-associated loci in a New Zealand discovery cohort (612 controls and 608 AAA cases), including four,* PCSK9* (1p32.3),* SORT1-CELSR2-PSRC1* (1p13.3),* APOA1* (11q23.3), and* LDLR* (19p13.2), which were all strongly associated with both phenotypes. Of these, 15 lead SNPs were associated with AAA with observed *P* values < 0.05. Three SNP associations, rs599839 on 1p13.3 (*SORT1-CELSR2-PSRC1*), rs4977574 on 9p21 (*CDKN2BAS1*), and rs4775049 on 15q21–23 (LIPC), had *P* < 0.1 in the same direction of association in a separate New Zealand validation cohort (1,766 controls and 713 AAA cases) and were followed up in another separate cohort, the Wellcome Trust Case Control Consortium (WTCCC) AAA cohort (5,605 controls and 1,286 AAA cases). Two SNPs remained significant following WTCCC AAA cohort validation. These were the 1p13.3* SORT1-CELSR2-PSRC1* rs599839, which represents a novel AAA association, and rs4977574 9p21* CDKN2BAS1*, which is in a previously reported AAA locus [44]. Replication in additional independent case-control cohorts demonstrated a consistent association with the G allele of rs599839 showing a protective effect within six of the 11 cohorts examined. In all of the 11 cohorts, the minor allele frequency (MAF) was lower in cases than in controls, and when all genotyped cohorts were analyzed together (7,048 AAA cases and 75,976 controls, pHet = 0.68), the meta *P* value was 7.2 × 10^−14^. Modeling for confounding interactions of concurrent dyslipidemia, heart disease, and other risk factors suggested that this marker is an independent predictor of AAA susceptibility. This study represents another important discovery of ours in the ongoing investigation of genetic markers in AAA.

### 3.4. Candidate Gene Studies for AAA

Candidate gene analysis is yet another study design and is the most common approach used to analyze the genetics behind AAAs [6, 8, 9]. Candidate genes are selected based on their biological function and their potential role in the disease pathogenesis. In Table 3 we summarize the most significant genetic associations with AAA identified in candidate gene and pathway-based studies.

In one of the candidate gene studies, we investigated the causal relationship between variants of lipoprotein(a) (*LPA*) and AAA [50]. Our interest in LPA stemmed from the work by others [58] who demonstrated an association between the alleles of two SNPs in the* LPA* gene, rs10455872 and rs3798220, and high plasma levels of LPA as well as CAD. We utilized DNA samples from 35 case-control series that included patients with ischemic stroke (effective sample size (*ne*) = 9,396), peripheral arterial disease (*ne* = 5,215), AAA (*n* = 4,572), venous thromboembolism (*ne* = 4,607), intracranial aneurysm (*ne* = 1,328), and CAD (*ne* = 12,716), as well as from 3,714 subjects with carotid intima. media thickness measurements. The main analyses assessed the association between the outcome variables and the total number of minor alleles of either SNP (C allele of rs3798220 or G allele of rs10455872). The total number of minor alleles (or LPA score) was associated with AAA (OR: 1.23; 95% CI: 1.11 to 1.36; *P* = 6.0 × 10^−5^). Given the substantial overlap between CAD and AAA, it is conceivable that the associations of the* LPA* variants with AAA are mediated through their association with CAD. To investigate this potential confounder, we reanalyzed the association between the* LPA* variants and AAA in cases with and without a history of CAD. After the exclusion of patients with CAD, the effect estimates for LPA score became lower for AAA (OR: 1.11; *P* = 0.16), suggesting a stronger association of the* LPA* score with atherosclerotic disease manifested in more than one vascular bed. Although this study had limitations, it demonstrated an association between two variants in the* LPA* gene and AAA. Further studies are necessary to confirm these intriguing results as the possible association between dyslipidemia and AAA remains controversial.

In one of our more recent AAA projects we began by investigating plausible and physiologic candidate genes on chromosome 19, which we had previously found to be linked to AAA in our affected relative pair-based linkage study [38]. The AAA linkage interval on chromosome 19q13 spans approximately 4 Mbp and contains over 100 genes [38]. In order to identify a smaller set of more relevant candidate genes, Gene Ontology [59] and the Kyoto Encyclopedia of Genes and Genomes (KEGG) [60] were used to identify nine genes with functions relevant to AAA pathogenesis. Eight of these genes were selected based on an annotated function in the immune system and one gene (*PEPD*) was included based on its potential for contributing to ECM remodeling in the AAA wall [61]. Using DNA samples from 394 cases and 419 controls, we genotyped 41 SNPs located in or around the nine candidate genes and found eight SNPs in three genes,* CD22* (2 SNPs),* PEPD* (5 SNPs), and* HAMP* (hepcidin antimicrobial peptide; 1 SNP), as being nominally associated with AAA (*P* < 0.05). When tested for genotypic association using logistic regression, six of these SNPs were nominally associated, two were not associated, and an additional SNP in* GPI* (glucose-6-phosphate isomerase) was identified; however, only the association with the SNP rs7248389 in the* PEPD* gene remained significant when correcting for multiple testing. Further analysis of the* PEPD* gene with DNA sequencing, however, failed to identify mutations responsible for AAA formation. These results demonstrate the difficulty in making educated guesses about potential targets in candidate gene studies.

### 3.5. Meta-Analysis of Genetic Association Studies on Polymorphisms in the Matrix Metalloproteinase Genes

A meta-analysis is a statistical technique that combines results from different studies in an effort to improve reliability. While most commonly applied to clinical studies it can also be used to improve the reliability of genetic studies. To this end, we performed a systematic review and meta-analysis of SNPs within the gene families of matrix metalloproteinases (MMPs) and their inhibitors (tissue inhibitor of metalloproteinases; TIMPs) and their association with AAA [54]. To accomplish this task, we performed a search of MEDLINE and EMBASE to identify studies assessing the association of SNPs in MMP and TIMP genes with AAA. We initially identified a total of 168 published studies. After more critical examination, only 13 studies were deemed suitable and were included in the meta-analysis. The combined population size of the 13 studies totaled 7,037 individuals (3,581 AAA case and 3,456 controls). Sample sizes ranged from 91 to 1,337 participants per study and represented a population of European ancestry from North America, Europe, and Australia. Studies varied with regard to inclusion criteria, screening for potentially undiagnosed AAA, distribution of conventional AAA risk factors, as well as matching cases and controls for AAA risk factors, age, sex, and smoking. All studies that reported differences in the prevalence of traditional AAA risk factors between AAA and control populations conducted multiple logistic regression analyses. Assessment of deviation from the Hardy-Weinberg equilibrium was stated in 10 studies; however, evaluation of genotyping error and reporting of appropriate rs numbers were evident in only four and five studies, respectively. Overall, 58 SNPs in 10 genes were examined. Of these, eight SNPs in eight genes (rs1799750,* MMP1*; rs3025058,* MMP3*; rs3918242,* MMP9*; rs486055,* MMP10*; rs2276109,* MMP12;* rs2252070,* MMP13*; rs4898,* TIMP1*; and rs9619311,* TIMP3*) were assessed commonly in at least three different sample populations and were included in the meta-analysis. Three SNPs were assessed in two studies and the remaining 47 SNPs were reported in only one study, mostly identified through sequencing of entire coding regions. This meta-analysis revealed that the 5A allele of* MMP3* rs3025058 (MAF = 48%) was significantly associated with AAA under dominant (OR = 1.48; 95% CI: 1.23 to 1.78; *P* = 3.95 × 10^−5^) and recessive (OR = 1.31; 95% CI: 1.10 to 1.56; *P* = 0.005) models of inheritance. Further investigations of this marker may improve our understanding of AAA pathogenesis and assist targeted AAA screening programs.

## 4. Novel Insights on AAA Pathobiology

### 4.1. The Role of Microorganisms in the Pathogenesis of AAA

**Figure 3:**
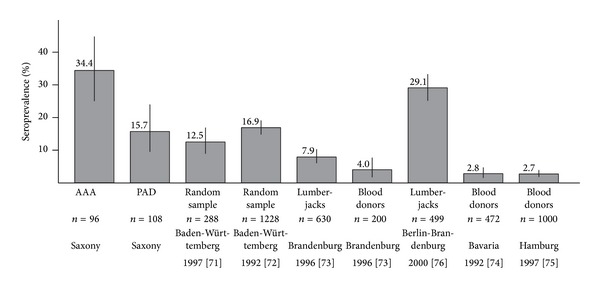
Seroprevalences for* Borrelia burgdorferi sensu lato* antibodies in different risk groups in Germany. Only studies with both ELISA and immunoblotting data were included. AAA, abdominal aortic aneurysm; PAD, peripheral artery disease. For details, see [70]. Reproduced with permission from [70].

Microorganisms, including* Chlamydia* [62–64],* Mycoplasma pneumoniae* [62],* Helicobacter pylori* [62],* human cytomegalovirus* (HCMV) [65],* herpes simplex virus* (HSV) [66], and different oral bacteria [67–69] may serve as possible triggers for the development of AAAs, but data remain inconclusive. We, too, were intrigued by a possible microbial trigger in AAA pathobiology and therefore studied whether* Borrelia burgdorferi sensu lato (sl)*, the microorganism responsible for Lyme disease, was involved in the etiology of AAAs [70]. Using a case-control design, we recruited 96 consecutive patients diagnosed with AAA using ultrasonography or CT (diameter ≥3 cm) at the Vascular Surgery Department, Technical University, Dresden, Germany. We collected venous blood samples from participants and analyzed them for antibodies against* B. burgdorferi* using an enzyme-linked immunosorbent assay (ELISA). Any positive result was confirmed by Western blot analysis. Among AAA patients, 34% were seropositive for* B. burgdorferi s.l.* antibodies, whereas only 15.7% of patients with peripheral artery disease (PAD) were seropositive (Figure 3; *P* = 0.003). In comparison, in the German general population, 3–17% are seropositive for* Borrelia* antibodies [71–75]. Among forest workers one study reported 7.9% seropositive rate [73] and another study 29.1% [76]. Our findings suggested a relationship between AAAs and* B. burgdorferi s.l.* We hypothesize that the underlying mechanism for* B. burgdorferi s.l.* in AAA formation is similar to that by the spirochete* Treponema pallidum*; alternatively, AAAs could develop due to induced autoimmunity via molecular mimicry or via similarities between* B. burgdorferi s.l.* proteins and aortic proteins. We recognize the limitations surrounding this small study and are planning a larger scale endeavor on a more diverse patient population.

### 4.2. Genome-Wide Microarray-Based mRNA Studies

An unbiased approach to study AAA pathogenesis at the molecular level is to carry out a genome-wide microarray-based mRNA or microRNA (miRNA) analysis to identify changes in mRNA and miRNA levels associated with AAA [7, 8, 33, 77, 78].* In silico* analysis can be used to classify the genes into functional groups and pathways. Another computational approach is to find transcription factor bindings sites in the genes with altered expression [79].

**Figure 4:**
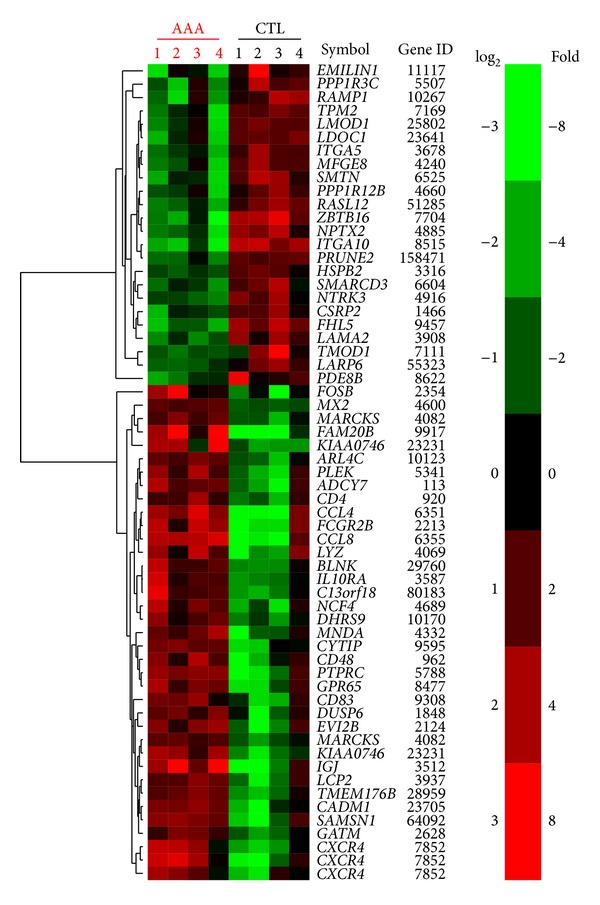
Heat map of the 57 differentially expressed genes found in two independent microarray studies for AAA. The heat map was produced by hierarchical clustering of the probeset data. Probesets for genes are represented by rows with the gene dendrogram at left. There are 61 probesets represented since there are redundant probesets on the Affymetrix microarray for some genes, for example, CXCR4, which is the gene represented by three lines at the bottom. Gene symbols and gene IDs on the right (found at http://www.ncbi.nlm.nih.gov/). Samples (4 AAA samples and 4 controls) are represented by columns. Green color indicates decreased expression in AAA and red color indicates increased expression in AAA.

We recently reported the results of our second microarray-based mRNA expression study on human AAA [80] and combined these findings with our previous study [81]. The combined analysis revealed 57 differentially expressed genes with consistent results in both studies (Figure 4). A total of 32 genes had increased and 25 decreased expression in AAA (Figure 4). The significance of the overlap between the two independent experiments was evaluated using Fisher's exact test. The odds ratio was 8.9 (95% CI: 5.6–14.3) and *P* < 2.2 × 10^−16^. We then selected 43 genes for follow-up studies with emphasis on genes not previously implicated in AAA pathobiology [80]. These genes represented a wide range of biological functional categories such as “*calcium signaling,*” “*cell development and differentiation,*” “*cell adhesion,*” and “*inflammatory response.*” The mRNA levels were significantly different between AAA and nonaneurysmal infrarenal aortic control samples in 38/43 (88%) genes tested using a RT-PCR-based PCR array method [80] and the direction of the change was the same seen in both microarray studies [80, 81]. Novel validated genes in AAA pathobiology included* ADCY7, ARL4C, BLNK, FOSB, GATM, LYZ, MFGE8, PRUNE2, PTPRC, SMTN, TMODI*, and* TPM2*. Considering that the results of two independent microarray studies and the PCR array were concordant, we are confident that these genes are differentially expressed in AAA and could contribute to AAA pathogenesis.

In another study, we focused on the complement cascade for further investigation [82]. Our previous microarray study [81] had shown that 13 (38%) of the 34 genes of the complement cascade arm of the “*complement and coagulation cascades*” KEGG pathway had significantly different mRNA expressions between AAA and control tissues. Five had increased and eight had decreased expression in AAA compared to the control tissues. Furthermore, follow-up studies showed strong staining of AAA tissue samples using an antibody specific to C2, one of the complement cascade proteins.* In silico* analysis of the promoter regions of the 13 differentially expressed complement cascade genes using Whole Genome rVISTA showed enrichment for binding sites for a transcription factor STAT5A when compared to the entire genome. To experimentally validate that STAT5A binds on the promoters of the complement cascade genes, chromatin immunoprecipitation followed by hybridization to high-density promoter arrays (ChIP-chip) was carried out using microarrays covering 10 kbp of the promoters of all known genes. STAT5A demonstrated binding to 6,297 distinct genes, 1,095 (17%) of which were differentially expressed in human AAA tissue (431 had increased expression and 664 had decreased expression). In the complement cascade genes, strong evidence for STAT5A binding was found on 10 of the 34 complement cascade genes and moderate evidence on nine complement cascade genes. In summary, this study provided multiple lines of evidence that complement cascade is important in AAA pathogenesis and that in human AAA the pathway is activated via the lectin and classical pathways.

Our microarray-based mRNA expression profiles [81] also formed the foundation for the study on HOX (homeodomain-containing) genes in AAA [83]. We had found that 78 HOX genes were expressed in the adult human aorta and that 31 of these had significantly different mRNA levels in AAA compared to control abdominal aortic tissues from an age-, sex-, and ethnicity-matched group of individuals. Ten of these 31 genes were so-called classic HOX genes. Interestingly, the mRNA levels of all of them were significantly decreased in AAA [83].* HOXA4* was the most significantly decreased HOX gene in human AAA tissue based on microarray results. We confirmed its decreased expression in an independent set of 12 AAA cases and 12 similarly aged controls using a real-time quantitative RT-PCR assay. We also detected* HOXA4* mRNA in cultured human aortic SMCs and aortic endothelial cells. Another intriguing finding was that HOX4A expression was lower in the abdominal than in the thoracic aorta. Abdominal aorta is known to be more susceptible for aneurysm formation. Downregulation of HOX genes could have several detrimental effects in the human aorta, since HOX genes have well-established roles in regulating cell proliferation and differentiation [83].

### 4.3. Genome-Wide MicroRNA (miRNA) Analysis for AAA

**Figure 5:**
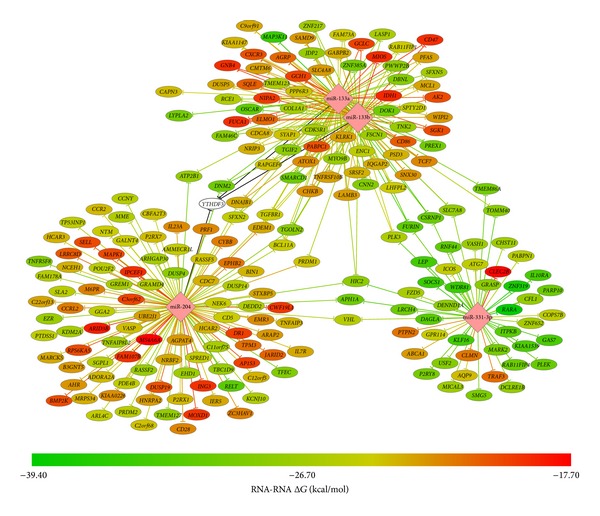
A Network of miRNAs, miR-133a, miR-133b, miR-331-3p, and miR-204, and their target genes. Each miRNA is predicted to regulate a large number of target genes (mRNAs). Bioinformatic analysis predicted that 222 genes with upregulated expression in AAA based on a prior microarray study [81] were targets of miR-133a, miR-133b, miR-331, or miR-204. The predicted minimum free energy of the miRNA and target mRNA hybridization from the program RNAhybrid version 2.1.is shown by the line and node color. Black lines for* YTHFD3* indicate that the 3′UTR sequence was not available from Ensembl BioMart, and the minimum free energy was not calculated. miR-30c-2* was not included because it is a miRNA* strand that is usually degraded and therefore nonfunctional. The figure is reproduced from [84].

miRNAs are small, well-conserved, noncoding molecules which can inhibit gene expression at the posttranscriptional level. Each miRNA is predicted to regulate a large number of target genes (mRNAs). We recently published our microarray-based genome-wide analysis of miRNA patterns in human AAA [84]. Five miRNAs (miR-133b, miR-133a, miR-331-3p, miR-30c-2, and miR-204) found to be downregulated in AAA were then validated with real-time quantitative RT-PCR and an independent set of samples. Extensive bioinformatic analyses were carried out to identify the mRNA targets of these miRNAs (Figure 5). A total of 1,836 potential target genes were found, 222 of which were significantly upregulated in our previously published mRNA expression study [81]. One of these targets was* MMP9* which has a well-established role in AAA pathogenesis [85].

### 4.4. Animal Models of AAA Pathogenesis

While it is our preference to use human AAA tissue in our studies, it can only be obtained during open repair operations and, therefore, studies on human AAA are restricted to end-stage disease with hugely altered aortic tissue architecture. Thankfully, several mouse models for aneurysms have been developed [8] providing an opportunity to study early stages of aneurysm development. The most widely used of them is the so-called angiotensin II- (AngII-) induced mouse model of aortic aneurysms in which mice lacking the apolipoprotein E gene receive an infusion of angiotensin II and develop vascular lesions including atherosclerosis, dissection, and dilatation of the suprarenal aorta. In a collaborative study with the team from the Nationwide Children's Hospital, we investigated the role of Notch signaling in aneurysm formation using this mouse model [86]. Notch 1 is a human gene that encodes a transmembrane receptor which plays multiple roles during development and cell differentiation. Notch 1 signaling was found to be activated in this AAA mouse model. Additionally, Notch 1 haploinsufficiency and pharmacological inhibition of Notch 1 signaling reduced the incidence of aortic aneurysms by preventing the accumulation of inflammatory cells to the aneurysm [86].

## 5. Electronic Medical Records and Biobanks: New Resources for Epidemiological and Genetic Research

GHS is an integrated, comprehensive health care delivery system that serves a large, stable, mainly rural population in North central and Northeastern Pennsylvania [22]. GHS is considered one of the most wired health care organizations in the USA and it has utilized a unified EMR since 1996 [22]. To facilitate research data mining and analysis, in 2008, GHS implemented a data warehouse system including the outpatient records of all patients seen by both primary care and specialty providers [22]. Another important initiative launched by GHS was the biobanking program called the MyCode project, which includes a central repository of patient samples (blood, DNA, serum, and tissue) that are linkable to EMR for broad research use in a manner that protects confidentiality of patient information [87].

To test our hypothesis that EMR can be utilized retrospectively to identify risk factors and that it is comparable to traditional epidemiological methods for risk factor assessment of a complex disease, we used AAA as a test case [88]. In this study approximately 900 AAA cases and over 14,000 controls were identified from the EMR at GHS and used to identify risk factors for AAA. We replicated the direction and magnitude of several known risk factors originally found in traditional epidemiological AAA studies [88]. This study demonstrated that EMR is a useful resource to assess risk factors and identify new associations.

GHS is one of the nine members of the electronic MEdical Records and GEnomics (eMERGE) network, a National Human Genome Research Institute- (NHGRI-) funded consortium which is developing methods and best practices for the utilization of the EMR as a tool for genomic research [89]. Each member in the eMERGE consortium has an EMR and a biobank and has proposed diseases or traits for which phenotyping algorithms [90] are being developed and genetic studies are carried out leading towards returning results to the patients and incorporating them into the EMR. We are fortunate that the GHS eMERGE team, along with others, has been assigned to lead the AAA-related efforts. Another important task for the eMERGE network is developing best practices and tools for patient and physician education.

At GHS the strategy for integration of genetic data into EMR is modeled on processes developed by the Clinical Transformation Team, a dedicated department devoted to creating information technology- (IT-) enabled improvements in patient care [22]. A “Care Gaps” strategy is used, in which clinical areas are evaluated to identify IT processes that can be incorporated into the EMR to improve patient care [22]. Care gaps looking to improve early diagnosis and screening for AAA are an ongoing effort of the Clinical Transformation Team.

The eMERGE network has generated data sets with available genome-wide and phenotypic data on a large number of individuals which provides an excellent resource for testing various hypotheses without a huge investment of funds for first generating the genotype data. For example, we contributed to a recent study investigating genetic pleiotropy for SNPs to identify underlying cellular mechanisms among diseases [91].

## 6. Outlook

The progress on our understanding of the clinical, genetic, and molecular factors contributing to the development, growth, and rupture of AAA has been remarkable in the past ten years. There are, however, still a large number of unsolved questions about AAA pathogenesis and how to translate the knowledge gained into better diagnostic and treatment options for AAA patients. Obtaining answers to these questions will require innovative, interdisciplinary approaches. It will be necessary to integrate information from epidemiological, genetic, molecular biology, and bioengineering studies on humans and animal models. It is more evident than ever that multicenter collaborations are needed for accomplishing these goals. Many of the findings described in this Outlook paper were discussed in two recent International Symposia on AAA which we helped to organize: (1) “*Abdominal Aortic Aneurysm: Epidemiology, Genetics, and Pathophysiology*” held on 20–22 October, 2011, at the Geisinger Clinic, Danville, Pennsylvania, USA [2] and (2) the “*3rd International Meeting on Aortic Diseases”* (IMAD3) held on October 4–6, 2012, in Liège, Belgium [5]. These disease-centered international conferences provide the ideal forum to engage experts from different disciplines into thought-provoking discussions and forward-thinking collaborations.

The vision we have for the future of AAA research is to expand the genetic and genomic studies to include larger samples sizes and different stages of the AAA development. Next steps will also include translating the basic science findings into diagnostic tests and medical treatment of AAA. The primary goal remains to prevent rupture by finding AAAs early to allow for safe surgical repair. It is likely that one day AAAs will be treated medically to slow their growth, helping to transform a surgical disease into a medical disease.

## Figures and Tables

**Table 1:** SAAVE act eligibility in 52 study patients with ruptured AAA at the time of presentation.

Variable	Total *n* (%)	Smoking history *n*	Family history of AAA *n*	SAAVE act eligible *n* (%)
Age (male)				
65–75	10 (19.2)	7	0	7 (13.5)
<65	7 (13.5)	5	0	0
>75	19 (36.5)	11	1	1 (1.9)
Female	16 (30.8)	13	1	1 (1.9)

Total				(17.3)

AAA: abdominal aortic aneurysm; smoking history: patients had smoked >100 cigarettes in their lifetime.

Modified from [21].

**Table 2:** AAA genetic loci discovered using genome-wide association studies.

Chromosomal location (study)	Nearest gene (gene symbol; gene ID)	Polymorphism	RAF	OR [95% CI]	Association *P* value^a^
3p12.3 [42]	Contactin-3 (*CNTN3*; 5067)	rs7635818	0.42	1.33 [1.10–1.21]	0.0028
9p21.3 [44]	CDKN2B antisense RNA 1 (*CDKN2BAS1*; 100048912)	rs10757278	0.45	1.31 [1.22–1.42]	1.2 × 10^−12^
9q33.1 [43]	DAB2 interacting protein (*DAB2IP*; 153090)	rs7025486	0.25	1.21 [1.14–1.28]	4.6 × 10^−10^
12q13.3 [45]	Low density lipoprotein receptor-related protein 1 (*LRP1*; 4035)	rs1466535	0.68	1.15 [1.10–1.21]	4.5 × 10^−10^
19p13.2 [41]	Low density lipoprotein receptor (*LDLR*; 3949)	rs6511720	0.88	0.76 [0.70–0.83]	2.1 × 10^−10^

RAF: risk allele frequency in population; OR: odds ratio; 95% CI: 95% confidence interval; rs number: unique identifier for each single nucleotide polymorphism (for more information, see http://www.ncbi.nlm.nih.gov/snp/).

Gene symbols and IDs are available from http://www.ncbi.nlm.nih.gov/gene/.

^a^ *P* values were taken from the first report demonstrating association with AAA and cited in the first column.

Modified from [4].

**Table 3:** Most significant associations with AAA discovered in candidate gene and pathway-based studies.

Gene name (study)	Functional class	Polymorphism	AAA cases *N*	Controls *N*	OR [95% CI]	Association *P* value^a^
*SORT1* [47]^b^	Lipid metabolism	rs599839	7,048	75,976	0.81 [0.76–0.85]	7.2 × 10^−14^
*IL6R* [49]	Inflammation	rs7529229	4,524	15,710	0.84 [0.80–0.89]	2.7 × 10^−11^
*LPA* [50]	Lipid metabolism	rs10455872 with rs3798220	4,572	33,520	1.23 [1.11–1.36]	6.0 × 10^−5^
*AGTR1* [51]^b^	Renin-angiotensin system	rs5186	1,226	1,723	1.60 [1.32–1.93]	1.1 × 10^−6^
*TGFBR2* [52]^b^	TGFB signaling	rs1036095	1,904	2,616	1.59 [1.23–2.07]	4.8 × 10^−4^
*TGFBR2* [52]^b^	TGFB signaling	rs764522	1,904	2,616	1.69 [1.28–2.25]	2.7 × 10^−4^
*ACE *[53]^b^	Renin-angiotensin system	rs4646994	1,415	1,677	1.35 [1.17–1.56]	<0.0001
*MMP3* [54]^b^	Degradation of ECM	rs3025058	1,258	1,406	1.48 [1.23–1.78]	3.95 × 10^−5^
*MMP13* [55]^b^	Degradation of ECM	rs2252070	800	843	1.37 [1.04–1.82]	NA
*MTHFD1* [56]	Methionine metabolism	rs8003379	423	423	0.41 [0.26–0.65]	<0.0001
*MTRR* [56]	Methionine metabolism	rs326118	423	423	0.47 [0.29–0.77]	0.003
*LRP5 *[57]	Lipid metabolism	rs3781590 rs4988300	423	423	2.16 [1.41–3.29]	<0.0001

OR: odds ratio; 95% CI: 95% confidence interval; ECM: extracellular matrix; TGFB: transforming growth factor beta; NA: not available. Gene symbols are available from http://www.ncbi.nlm.nih.gov/gene/.

^a^ *P*-values were taken from either a meta-analysis or the largest report demonstrating association with AAA and cited in the first column.

^b^Meta-analysis.

Modified from [4].
